# Intermolecular Interactions in Crystal Structures of Imatinib-Containing Compounds

**DOI:** 10.3390/ijms21238970

**Published:** 2020-11-26

**Authors:** Anna V. Vologzhanina, Ivan E. Ushakov, Alexander A. Korlyukov

**Affiliations:** 1 A. N. Nesmeyanov Institute of Organoelement Compounds, Russian Academy of Sciences, 28 Vavilova Str., 119991 Moscow, Russia; vologzhanina@mail.ru (A.V.V.); f0rbmen@gmail.com (I.E.U.); 2 Higher Chemical College of Russian Academy of Sciences, D. M. Mendeleev University of Chemical Technology of Russia, Miusskaya sq. 9, 125047 Moscow, Russia

**Keywords:** API, DFT calculations, likelihood of H-bond formation, molecular Voronoi polyhedron

## Abstract

Imatinib, one of the most used therapeutic agents to treat leukemia, is an inhibitor that specifically blocks the activity of tyrosine kinases. The molecule of imatinib is flexible and contains several functional groups able to take part in H-bonding and hydrophobic interactions. Analysis of molecular conformations for this drug was carried out using density functional theory calculations of rotation potentials along single bonds and by analyzing crystal structures of imatinib-containing compounds taken from the Cambridge Structural Database and the Protein Data Bank. Rotation along the N-C bond in the region of the amide group was found to be the reason for two relatively stable molecular conformations, an extended and a folded one. The role of various types of intermolecular interactions in stabilization of the particular molecular conformation was studied in terms of (i) the likelihood of H-bond formation, and (ii) their contribution to the Voronoi molecular surface. It is shown that experimentally observed hydrogen bonds are in accord with the likelihood of their formation. The number of H-bonds in ligand-receptor complexes surpasses that in imatinib salts due to the large number of donors and acceptors of H-bonding within the binding pocket of tyrosine kinases. Contribution of hydrophilic intermolecular interactions to the Voronoi molecular surface is similar for both conformations, while π...π stacking is more typical for the folded conformation of imatinib.

## 1. Introduction

**Scheme 1:**
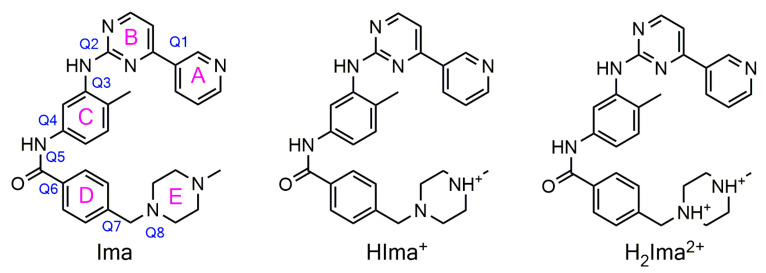
Schematic representation of imatinib in neutral (Ima) and protonated (HIma^+^, H_2_Ima^2+^) forms. Letters A–E indicate rings, and Q1–Q8 denote torsion angles about corresponding single bonds.

Chronic myelogenic leukemia (CML) is caused by expression of a single oncoprotein resulting from the fusion of proteins involved in multiple signaling pathways [1]. The fusion causes leukemic transformation [2] due to unregulated tyrosine kinase activity [3]. The first therapeutic agent to treat CML through inhibition of fusion is imatinib [4,5] (Ima, commercially available as *Gleevec*, Scheme 1), which acts against a very limited set of tyrosine kinases [6] and some transmembrane transporters [7]. A series of structural studies elucidated that imatinib specifically binds to an inactive Abelson tyrosine kinase domain characteristic for this gene through numerous hydrogen bonds [8,9]. Besides, hydrophobic C-H…π and π…π interactions were experimentally observed in imatinib–kinase complexes characterized using X-ray diffraction. However, the binding pocket of Abelson tyrosine kinase allows imatinib to realize only a limited number of molecular conformations of this flexible molecule, while another molecular environment can stabilize another molecular conformation through another set of intermolecular interactions. Particularly, comparison of imatinib conformations observed in single crystals and ligand-protein complexes reported in 2012 allowed Golzarroshan et al. to propose that although this flexible molecule contains eight single bonds, it realizes in crystals two main conformations, an extended with the pyridylpyrimidine moiety in trans position towards the methylbenzene ring and a folded with the pyridylpyrimidine moiety cis situated to the methylbenzene ring [10]. In vacuo molecular dynamics simulations carried out using the OPLS-2005 force field also demonstrated stability of these two conformations in solution.

Since 2012, a number of other crystal structures were additionally reported. Among them are crystal structures of two polymorphs of imatinib mesylate, that is the active pharmaceutical ingredient (API) of the therapeutic agent against CML, where the imatinib molecule adopted different conformations. However, correlations between various crystalline environments, intermolecular interactions, and imatinib conformations have not been studied before. Analysis of correlations would allow one to reveal the most abundant intermolecular interactions that tend to appear in all crystal structures regardless of molecular conformation. Another task of this comparison is to estimate the effect of a crystalline environment and molecular conformations on intermolecular interactions’ appearance. Similar types of interactions are present in both small-crystal structures and proteins (besides well-known hydrogen bonds, such examples as carbonyl…carbonyl [11], carbonyl…halogen, nitrile…sulfur, and some other [12] interactions can be mentioned). However, it is known that the average O/N-H donor/acceptor ratio in proteins is much higher than that in small organic structures, which makes some hydrogen bonds (for example, with organic fluorine) more likely in protein–ligand complexes than in small-molecule structures. Thus, we investigated the likelihood of H-bond formation in imatinib salts and complexes using the H-bonding propensity tool [13] implemented within the Mercury package [14]. This approach allows ranking of various donors and acceptors of H-bonding based on data knowledge about occurrences of various synthons extracted from the Cambridge Structural Database (CSD). Although it was proposed for prediction of polymorphism induced by variation of the system of H-bonds, the tool demonstrated effectiveness of prediction of cocrystal and solvate formation and nonformation for some drug molecules [15,16,17,18]. The procedure of analysis allows ranking the most and the least likely pairs of donors and acceptors as described in [13,19].

Qualitative and semiquantitative analysis of all intermolecular interactions including very weak and forced ones can be carried out within the Voronoi tessellation of a crystal space. Within this approach, all points within an atomic domain are closer to the inner atom than to any external one, and a molecular Voronoi polyhedron (MVP) can be constructed of Voronoi polyhedra of all atoms the molecule consists of [20]. The molecular Voronoi surface is faceted by the faces of atomic Voronoi polyhedra, and a pair of atoms is considered to form an intermolecular interaction if they share any molecular surface. This is a convenient method to estimate molecular coordination number [20], molecular size [21,22], and the contribution of various types of intermolecular interactions to a molecular surface [23,24,25,26]. Recently, some of us demonstrated similarity of contribution of hydrophobic and hydrophilic interactions of abiraterone in small-crystal structures and ligand–receptor complexes using this approach [27].

Herein we report analysis of molecular flexibility using Density Functional Theory (DFT) calculations of rotation potentials for pure imatinib and its protonated forms, and comparison of intermolecular interactions of imatinib in different conformations and crystalline environment. H-bonds in salts and ligand-receptor complexes are compared. Contribution of various types of intermolecular interactions to the molecular surface of imatinib using the MVP approach is studied, and applicability of MVP-based analysis of intermolecular interactions to flexible molecules is demonstrated.

## 2. Results

### 2.1. Conformational Analysis of Imatinib

**Figure 1:**
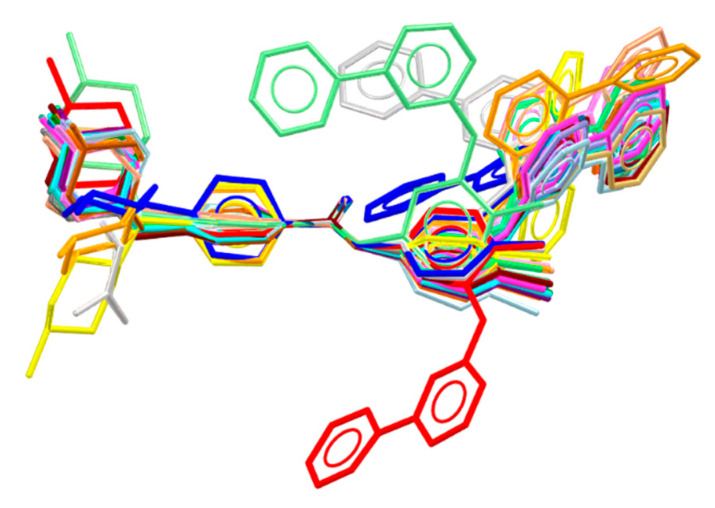
Conformations of imatinib in studied compounds. Four superimposed atoms belong to the amide C_Ph_C (O)N fragment. Compounds with CSD codes DUNTIQ (red, [30]), XAVTOF (orange, [31]), XAVTOF02 (yellow, [31]), XEJLUW (light green, [32]), and PDB codes 1IEP (green, [33]), 1OPJ (purple, [9]), 1T46 (violet, [34]), 1XBB (light grey, [35]), 2HYY (light blue, [36]), 2OIQ (cyan, [37]), 3FW1 (blue, [29]), 3GVU (sienna, [38]), 3HEC (peach pink, [39]), 3K5V (light emerald, [40]), 3MS9 (lilac, [41]), 3MSS (teal, [41]), 3OEZ (brown, [42]), 3PYY (light brown, [43]), 4CSV (magenta, [28]), 6HD4 (grey [44]), 6JOL (sea-green, [45]), 6NPE (orange-brown, [46]) are depicted.

In Figure 1, conformations of imatinib in 29 X-rayed crystals are compared with superimposed amide fragments. For example, the imatinib molecules depicted in magenta (code 4CSV in the Protein Data Bank, PDB, [28]) and blue (PDB code 3FW1 [29]) correspond to the extended and folded conformations, respectively. Few crystal structures reported later in general support the conclusion of Golzarroshan et al. [10] about the stability of two conformations and prevalence of the extended conformation in ligand–receptor complexes, as only two of the ligand–receptor complexes (PDB codes 1XBB and 3FW1 [29]) contain ligand in the folded conformation. However, imatinib salts reported to date also demonstrate a variety of molecular conformations in the folded conformation (Figure 1). 

**Figure 2:**
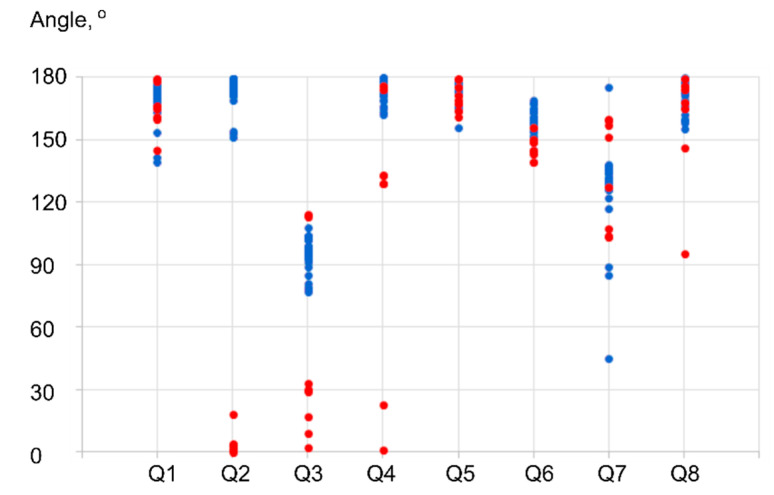
Modules of Q1–Q8 torsion angles of imatinib in (blue) extended and (red) folded conformations in 43 molecules found in 29 crystals.

Values of Q1–Q8 torsion angles that describe rotation of functional groups and rings along single bonds are listed in Table S1 (Electronic Supporting Information) and visualized in Figure 2. Based on these data, the most rigid fragments of imatinib include the nearly coplanar disposition of the pyridyl (A) and the pyperazine (B) rings (angle Q1), and the nearly coplanar benzamide group and benzene ring (D) (angles Q5 and Q6). Besides planarity, the rigidity of pyridylpyrimidine moiety manifests itself through stability of nitrogen atom positions; the nitrogen atom of the pyridyl ring is always situated cis to the N3 atom of the pyrimidine ring. The largest variety of values is observed in both conformations for the Q7 angle corresponding to rotation of the piperidine ring (E) along the C-C bonds of the methylene-group. The Q2 and Q3 torsion angles that denote the rotation of the pyridylpyrimidine along the C-N bonds of the amino group also significantly vary, but these depend on the molecular conformation. Corresponding values for the extended and folded conformations form nonoverlapping sets and, thus, denote the conformation. These results are in accord with our calculations of rotation barriers for the Q1–Q8 angles.

The rotation potentials for Ima and HIma^+^ molecules were obtained from quantum chemical calculations at the B3LYP/6-31G(d) level of theory (see Section 4 for details). According to these calculations, the potentials of rotation of both molecules are approximately the same, except for rotation around the N-C bond, which is close to the protonated fragment (E). The rotation potentials of the angles Q1–Q8 are shown in Table 1, and the dependences of the energy by the dihedral angles Q1–Q8 are depicted on Figures S1–S8 (Electronic Supporting Information). Rotation along the (O)C-C_Ar_ (Q4) and along the C(pyridyl)-C(pyrimidine) bonds is characterized by the lowest rotation potential, followed by the potentials of rotation along C-CH2 bonds with pipyridine. The barriers of rotation along N–C bonds are higher except for the N(amide)-C_Ar_ bond. Experimentally observed geometry in general follows the calculations giving a variety of molecular conformations, mainly due to disposition of pipyridine fragment and rotation along the N(amide)-C_Ar_ bond (Figure 1). Experimentally observed Q1–Q2 and Q4–Q8 values are also remarkably close to the most stable values. Only Q3 experimental values strongly deviate from the values corresponding to the stable conformation. The most stable geometry of both Ima and HIma^+^ molecules corresponds to the extended conformation. For an uncharged Ima molecule, a folded conformer is 5.11 kcal/mol less favorable than an extended one. The situation is quite different in the case of protonated charged conformers; the difference in their total energy is negligible (0.15 kcal/mol only). 

To sum up, imatinib belongs to flexible molecules with low barriers of rotation along the amide and methylene groups. The particular conformation depends on the interplay of inter- and intramolecular interactions affected by the crystalline environment. However, none of the studied compounds realizes strong intramolecular bonding, and all functional groups of the API remain available for intermolecular bonding only. Among all intermolecular interactions, competition of functional groups in the crystalline environment to form the strongest of these, the hydrogen bonds, can be numerically characterized by calculation of the likelihood of H-bond formation (Section 2.2). The role of the other types of interactions in stabilization of the particular conformation is discussed in Section 2.3 in terms of their contribution to the total surface of the molecular Voronoi polyhedron.

### 2.2. H-Bonding in Crystals of Imatinib-Containing Compounds

The imatinib molecule contains six functional groups (tertiary amines of piperidyl, secondary amine between B and C rings, three types of aromatic nitrogen atoms and amide) potentially able to form H-bonds that contain in total two donors (the nitrogen atoms of secondary amine and of amide) and six acceptors of H-bonding. Thus, pure solvent-free Ima can theoretically realize 12 different types of hydrogen bonds. These bonds are ranked with good discrimination (area under ROC curve is equal to 0.81) as listed in Table 2. Among them, the amine…pyridyl, amide…pyridyl, amide…amide, and amine…amide NH…O hydrogen bond are the most likely to occur, and the NH…N bond with nitrogen atoms of piperidyl or secondary amine are the least likely to form. Unfortunately, none of the X-rayed solid forms contains neutral Ima, while HIma^+^ contains an additional donor of H-bond (tertiary ammonium), and counterions that also contain functional groups. However, H-bonding interactions in ligand–receptor complexes can be roughly estimated using functional groups of HIma^+^ and water molecules, as receptors are also to use amide groups to form hydrogen bonds with the API (but contain also other donors and acceptors of H-bonding). Area under ROC curve of 0.79 indicates acceptable discrimination for the logistic model. The results of calculations indicate that, in general, water molecules and tertiary ammonium of piperidinium are better donors of H-bonding than secondary amine and amide groups. However, the difference between propensity of H-bond formation with water or pyridine, amide or pyrimidine as acceptors is rather low, which explains the absence of hydrates among salts, as it was analyzed in ref. [47]. The nitrogen atom of pyridine and oxygen atom of amide remain the best acceptors of H-bonding followed by water molecules.

**Figure 3:**
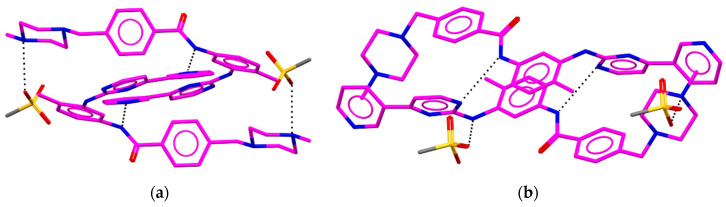
Fragment of H-bonded motifs in crystals of (**a**) (HIma)(O_3_SCH_3_)·MeOH·H_2_O, CSD code XEJLUW [32] and (**b**) α-form of (HIma)(O_3_SCH_3_), CSD code XAVTOF [31]. H-bonds are dotted. Hydrogen atoms are omitted for clarity.

Experimentally observed synthons in general follow the trends predicted with the H-bonding propensities tool. In crystals of small molecules, the best donors of H-bonding (tertiary ammonium and secondary amine) take part in intermolecular interactions with oxygen atoms of anions (mesylate, picrate) that are supposed to be better acceptor groups than any group of HIma^+^. The amide group interacts with one of the most expected acceptor groups of the API, the nitrogen atom of pyridine or pyrimidine. For both conformations, the resulting H-bonded motif is an H-bonded dimer of HIma^+^ molecules (Figure 3a,b).

**Figure 4:**
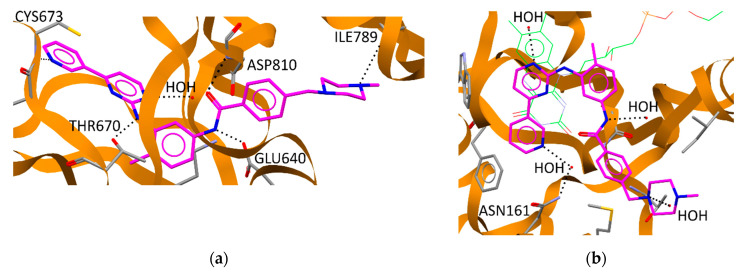
H-bonded motifs in crystals of imatinib complexes with (**a**) tyrosine kinase (PDB code: 1T46 [34]) and (**b**) quinone oxidoreductase (PDB code: 3FW1 [29]). H-bonds are dotted. Hydrogen atoms are omitted.

In ligand–receptor complexes, the number of donors and acceptors of H-bonding in the API environment surpasses the number of H-bond donors and acceptors in the ligand. As a result, all H(N) atoms of imatinib are involved in hydrogen bonding (Figure 4a), and the only acceptor group typically not bonded with any donor of an H-bond is the tertiary amine of the piperidinium group that is in accord with the expected likelihood of H-bond formation (Table 2). Hydrogen atoms of tertiary ammonium in Ima^+^: receptor complexes with tyrosine kinases are involved in interactions with an amide group of the receptor. The secondary amine interacts with an OH group of THR residue. The H(N) atom of the amide group forms an H-bond with a carboxylic group of GLU residue. Note that an amide or a carboxyllic group is expected to be a slightly better acceptor of H-bonding than water molecules, and none of the H-bonding donors in the Ima^+^ complexes with kinases interacts with water molecules.

However, in spleen tyrosine kinase (SYK) [35] and quinone oxidoreductase NQO2 [29], steric constraints prevent imatinib from binding in an extended form. Moreover, the binding pocket of NQO2 is hydrophobic, and donor groups of API take part in hydrogen bonding with water molecules only (Figure 4b). The H-bonding between SYK and imatinib is also limited with N-H…O and N-H…N interactions of ALA451 residue of kinase, and secondary amine and pyrimidine groups of API, and the pyridine is H-bonded with a water molecule, while the rest of the intermolecular interactions are hydrophobic. In the imatinib-NQO2 complex, there are three interactions between the ligand and water molecules, and one of them acts as a bridge between the pyridyl nitrogen and ASN to form a so-called “masked synthon” [48]. 

Thus, mutual disposition of amide groups in the binding pocket of tyrosine kinases ABL, CDK4, KIT, and SRC allows the extended form of imatinib to realize all expected hydrogen bonds of amine, amide, and ammonium groups, as well as of nitrogen atoms of pyridyl and pyrimidine rings. However, in complexes with SYK and NQO2, the same molecule is stabilized within a binding pocket mainly through hydrophobic interactions, thus, contributions of hydrophobic and hydrophilic interactions to the total packing and the effect of molecular conformation on the role of hydrophobic interactions are of interest. 

### 2.3. Contribution of Various Types of Intermolecular Interactions to the Voronoi Surface of Imatinib

**Figure 5:**
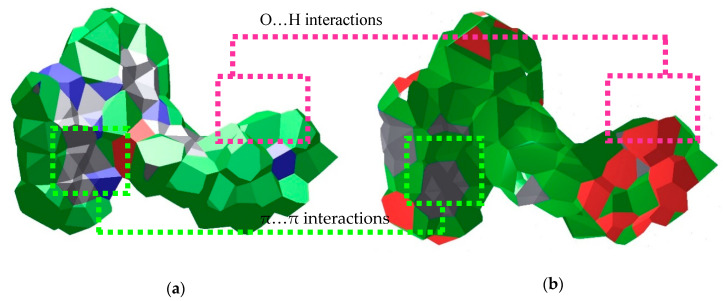
Molecular Voronoi polyhedron of imatinib in complex with the quinone oxidoreductase NQO2 (PDB code 3FW1 [29]) colored in accord with the nature of (**a**) inner, imatinib and (**b**) outer, receptor atoms. Color code: C-gray, H-green, N-blue, O-red. The π…π interactions in the region of pyrid-3-yl and the O…H interactions in the region of piperidine rings are additionally marked.

In Figure 5, a molecular Voronoi polyhedron of the imatinib molecule in complex with the quinone oxidoreductase NQO2 (PDB code 3FW1 [29]) is depicted. The MVP surface can be colored in accord with the nature of inner (Y, imatinib, Figure 5a) and outer (Z, receptor, solvent, or neighboring molecule, Figure 5b) atoms, and all the types of Y…Z interactions observed for a molecule in a particular crystalline environment can be estimated. Visualization of the ligand environment is a convenient tool to analyze steric clashes in ligand/receptor complexes and the positions of a ligand which can be modified to affect the selectivity of ligands.

MVP of 21 independent HIma^+^ molecules in complexes with receptors, and of 6 salts were reconstructed; their volumes, surfaces, and contribution of various types of intermolecular interactions to the surface were calculated. The list of compounds is given in Appendix A; unfortunately, the rest of the imatinib complexes stored in the PDB contain voids with unresolved content. For compounds containing voids, MVP of Ima complexes could not be calculated. Numerical values of V_MVP_, S_MVP_, and partial contributions of various types of interactions to the total area are listed in Table S2 (ESI). Normal probability plots (Figure S9, ESI) and analysis of skewness and kurtosis (Appendix B) provide statistical evidence that the imatinib volume and molecular area are normally distributed despite various molecular conformations and environment. For comparison, similar analysis of the second normalized moment (G_3_), the descriptor of molecular sphericity [49], indicates that the extended and folded conformations of imatinib have different G_3_ values that do not form a normal distribution. G_3_ values for the former are higher than those for the latter (0.257(7) and 0.181(30)) as could be expected for a molecule with the larger aspect ratio (G_3_ = 0.077, corresponds to a sphere). The mean volume of the molecular Voronoi polyhedron of imatinib is equal to 676.7(511) Å^3^, and the total molecular area of MVP is equal to 661.1(373) Å^2^. Although the molecular volume of imatinib in salts is typically lower than that in ligand-receptor complexes, that can be referred to unresolved disorder of peptide chains [27] or missing water molecules, these values are still within a confidence interval at the *p* = 0.95 confidence level. All experimentally observed molecular volumes and molecular areas lie within the confidence interval at *p* = 0.95 equal to, respectively, [576.6; 776.9] Å^3^ and [587.9; 734.3] Å^2^. Thus, all molecular volumes and areas can be considered as coincident, and the role of various intermolecular interactions in stabilization of a molecular conformation can be estimated using one-way analysis of variance of their partial contributions to the total area of MVP.

For all molecules under discussion, hydrophobic H…H, C…H, C…C, and C…N and hydrophilic O…H and N…H interactions were found. Besides, all compounds taken from the Protein Data Bank realize S…H, C…O, and O…O interactions. Depending on chemical composition of a complex or salt, C…S, N…S, N…O, N…N, and Cl…H interactions can also appear. Thus, one can evaluate either the role of hydrophilic/hydrophobic interactions or of a particular interaction type found in all compounds using the Voronoi approach. Unexpectedly, despite variation in the chemical environment of the imatinib molecule in complexes and salts, c.a. ¾ of the molecular area goes to hydrophobic interactions. Their partial contribution varies in a narrow range and is equal to 75.8(1.5)%. Confidence interval at *p* = 0.95 is equal to [72.9; 78.6]%, and only two crystal structures, namely, {RAYQEP} and {DUNTIQ} both containing 2,4,6-trinitrophenolate anion, do not fit within the confidence interval either at *p* = 0.95, or at *p* = 0.99 and 0.999. This may be related with the inclination of nitro groups to take part in O…H bonding, or the low number of hydrogen atoms in the 2,4,6-trinitrophenolate anion.

The results of analysis of variance for π…π interactions are listed in Table 3; those for H…H, C…H, C…N, O…H, and N…H interactions and hydrophobic/hydrophilic interactions in general are listed in Tables S3–S5 (Electronic Supporting Information). Although these are strong hydrogen bonds that are typically assumed to stabilize an unfavorable conformation (the extended one for imatinib), there is no statistically significant difference in partial contribution of O…H interactions to the total molecular surface for two different conformations. Moreover, contribution of hydrophilic interactions in sum to the molecular area correlates with the molecular conformation not above 30%, while the corresponding value of R^2^ for the hydrophobic interactions exceeds 70% (Table S3). Of all hydrophobic interactions, the role of π…π stacking interactions is the most prominent. As it follows from Table 3, the contribution of stacking interactions to the molecular area from pyridylpyrimidine (C…N interactions) and benzyl (C…C interactions) rings in two conformations remarkably differs (difference in the molecular area that goes to π…π interactions correlates with a conformation with R^2^ = 0.98). Absolute values of the area that goes to C…C and C…N interactions are rather low, 5.9 [1.6; 10.2] Å^2^ for the extended conformation and 29.3 [23.2; 35.3] Å^2^ for the folded conformation, or 1 and 4% of the mean molecular area, however, one should take into account that the Voronoi partitioning underestimates the contribution of π…π interactions to the total energy of crystal packing and to the molecular surface [50,51,52]. Among hydrophilic interactions, some correlation between contribution of N…H interactions to the molecular area and molecular conformation was found (R^2^ = 0.49). This fact may be with experimental observation that nitrogen atoms of pyridine, pyrimidine, and pypiredyl more often act as acceptors of H-bonding in ligand–receptor complexes. However, correlation between the absence of strong H-bonding with the oxygen atom of the amide group in salts with the folded imatinib conformation and their presence in ligand–receptor complexes is absent (R^2^ = 0.04).

## 3. Discussion

The concept of a molecular surface and volume is of considerable value in modern chemistry and crystallography. Their application include understanding of intermolecular interactions, analysis of complementarity of host-guest and drug-receptor compounds, understanding bulk physical properties, and visualization of some properties (electrostatic potential, electron density, nature of inner and outer atoms, et cetera) by mapping on the surface. In solids, the surface and volume can be obtained and visualized within the Quantum Theory of Atoms in Molecules (QTAIM) [53], the Hirshfeld surface approach [54], and the Voronoi tessellation of crystal space [55]. The advantages of the latter approach include simple and fast calculation (as compared with the QTAIM approach), wide range of investigation objects (from monoatomic compounds to biomacromolecules), additional atomic and molecular descriptors of their shape, and absence of any van der Waals radii during calculations. The Voronoi tessellation has been previously applied for analysis of protein volumes and packing [56,57,58] and interactions between macromolecules [59,60], as well as estimation of protein data quality [61]. Some other applications for study of macromolecules are reviewed in refs. [62,63]. Herein we report comparison of molecular volumes of a given molecule in salts and ligand–receptor complexes estimated by means of the Voronoi tessellation. It was demonstrated for the first time that although the volume of the imatinib molecular Voronoi polyhedron in complexes is slightly larger than in salts, this difference is statistically insignificant. Thus, constancy of molecular volume in salts and complexes despite conformation variation can be used for estimation if a binding pocket of a receptor fits in a molecule by comparison of free space within a pocket and of molecular volume in pure solid, salt, or cocrystal. A similar strategy was previously applied for analysis of cucurbituril host–guest compounds [64]. During this study we also found that V_MVP_ and G_3_ were sensitive to the presence of voids in crystals of ligand–receptor complexes. Corresponding V_MVP_ and G_3_ values of imatinib in such solids exceeded confidence intervals even at *p* = 0.99. While estimation of protein data quality is based on comparison of volumes of protein residues with tabular data [61], the volume of ligands can also be used for verification of refinement of ligand-receptor complexes to find missing solvent molecules or ions.

Note also that the molecular Voronoi polyhedron represents well the molecular domain bounded by the zero-flux surface [50,65,66,67]. Thus, analysis of the closest environment mapped on the molecular Voronoi polyhedron allows excluding protein residues not involved in bonding with the ligand, and finding molecular regions that can be modified to increase or decrease overall energy of ligand-receptor bonding (see analysis of abiraterone environment in ref. [27]). In-depth analysis of intermolecular interactions within the Voronoi approach utilizes the idea that contribution of various types of interactions to the total molecular surface is in proportion with contribution of intermolecular interactions to the total packing energy [50,65,66,67]. In refs. [50,51,52] it was demonstrated that this approach underestimates the role of π…π interactions as the Voronoi partitioning neglects the shift of interatomic zero-flux surface from carbon to hydrogen atoms, however, it typically represents well contributions of hydrophobic and hydrophilic interactions to the total packing energy. In this work we found that contribution of hydrophobic and hydrophilic interactions remains unchanged for all imatinib conformations. On one hand, this result is in accord with the observation that the total electrostatic interaction energy of another flexible molecule, bicalutamide, in complexes with androgen receptor was comparable with the electrostatic lattice energy of the monoclinic bicalutamide polymorph despite differences in molecular conformations [68]. On the other hand, we expected to find that the contribution of O…H and N…H hydrogen bonds to the total molecular surface would increase with an increase in the number of strong hydrogen bonds. Constancy of area that goes to O…H and N…H interactions demonstrated another shortcoming of the Voronoi partitioning; here, that weak C-H…O and C-H…N interactions cannot be distinguished from stronger N-H…O, N-H…N, O-H…O, and O-H…N hydrogen bonds. Another possibility is that the total molecular conformation, shape, and surface area are determined by the interplay of all interactions in a given crystalline environment rather than particular H-bonded interactions. Thus, additional analysis of energy of intermolecular bonding of imatinib (or other molecules) in salts and complexes should be carried out to check the hypothesis that a high number of hydrogen bonds can stabilize an unfavorable conformation. Another shortcoming of the Voronoi approach is the need to calculate positions of hydrogen atoms that are not always obvious for residues and water molecules. At the same time, we found high correlation between molecular conformation and contribution of π…π interactions to the total area. This result is in accord with the conclusion given in ref. [35] about hydrophobic character of the binding pocket surface of SYK. Thus, we demonstrated for the first time that the molecular Voronoi polyhedra can be used for analysis of variance related with some molecular descriptors on one hand, and contribution of some intermolecular interactions (even very weak ones) on the other. In this paper the effect of molecular conformation was studied, although intermolecular interactions in series of homologues, neutral, and charged species can also be compared in such way.

## 4. Materials and Methods

The Cambridge Structural Database (CSD; [69]) and the Protein Data Bank (PDB; [70]) contain crystal structures of imatinib in uncharged Ima and protonated HIma^+^ and H_2_Ima^2+^ forms. For conformational analysis, six crystal structures were taken from the CSD-2020.1.1, and 21 Ima-receptor complexes were selected from the PDB (PDB codes are listed in Appendix A). We selected PDB complexes with resolution better than or equal to 3.0 Å, and working R-factors better than 0.25, measured at 80–120 K.

Analysis of intermolecular interactions was carried out only for crystals containing imatinib in the protonated HIma^+^ form. Forms α and β of imatinib mesylate [31] studied and room temperature were excluded from consideration due to high disorder. For imatinib picrate [10], imatinib mesylate [31], and its solvates [32], only one of two disordered positions was analyzed. For Ima–receptor complexes, the total charge of ligand was proposed based on presence and the charge of counter anions, while position of additional proton can be proposed based on the system of H-bonds, and disordered structures were excluded from analysis. After applying these criteria, crystal structures containing HIma^+^ bound to the ligand-binding domain of mouse kinase (1IEP, 1OPJ, 3K5V, 3MS9, 3MSS), human tyrosine kinase (1T46, 2HYY, 3GVU, 3PYY, 4BKJ, 6JOL, 6NPV), chicken kinase (2OIQ, 3OEZ) were used for further analysis. We used the Hermes algorithm [71] to add hydrogen atoms to water molecules, protein residues, and ligands in order to optimize the hydrogen bond networks. Intramolecular connectivity and intermolecular interactions of all crystal structures were then derived using the Voronoi approach with the ToposPro suite [72].

The rotational potentials were calculated from relaxed scans of potential energy. All quantum chemical calculations were carried out with B3LYP/6-31G(d) level of theory using the “ModRedundant” feature of the Gaussian 09 program [73] using standard settings and convergence criteria (Grid = Fine, 10^−8^ a.u. for each SCF step). The calculations started from the search of the most stable conformers of neutral Ima and its protonated form using the coordinates taken from CCDC. Optimized structures of conformers were tested afterward to correspond to the global minima by calculations of Hessian matrix. After that, the relaxed potential energy scans for most of the stable conformers were carried out using the following procedure: the values of the Q1–Q8 angles were gradually changed in interval 0–360° with stepsize 1° (except for Q1, where the angle rotation was 180 degrees). The rest of the molecular degrees of freedom were optimized at each step.

## 5. Conclusions

Flexibility of the imatinib molecule was numerically characterized using DFT calculations; for both Ima and HIma^+^, the extended conformation was found to be more stable than the folded one by 5.11 and 0.15 kcal/mol. Intermolecular interactions of the imatinib molecule in different conformations observed in ligand-receptor complexes and in salts were studied. The likelihood of formation of hydrogen bonds was estimated and contribution of various types of intermolecular interactions to the total molecular surface was calculated. The following results were obtained:

(i) Tertiary ammonium and secondary amine are the best donors of H-bonding, and tertiary amine of piperidyl is the worst. Experimentally observed synthons in general follow the trends predicted with the H-bonding propensities tool in both salts and ligand–receptor complexes. However, the hydrophilic surface of the binding pocket in receptors allows realizing H-bonds with almost all donors and acceptors of H-bonding in the imatinib molecule except for the least likely one, while in salts only the most likely H-bonds were found. Similar likelihood of H-bond formation between water or amide may be the reason for the absence of hydrates in X-rayed salts.
(ii) Constancy of volume and total area of the molecular Voronoi polyhedron of imatinib in different crystalline environments and in various conformations was demonstrated. Their mean values were equal to 676.7(511) Å^3^ and 661.1(373) Å^2^, and confidence intervals at *p* = 0.95 were [576.6; 776.9] Å^3^ and [587.9; 734.3] Å^2^.
(iii) For all compounds except two picrates, 75.8(15)% of the total molecular area goes to hydrophobic interactions. Although the total number of strong hydrogen bonds of the imatinib molecule in the extended conformation is higher than that of the molecule in the folded conformation, the molecular area that goes to all hydrophobic interactions, or to all O…H and N…H, or to strong hydrogen bonds for both conformations is nearly the same. At the same time, contribution of π…π stacking interactions to the molecular area of imatinib molecules in the folded conformation is more prominent than to the area of molecules in the extended conformation.

Methodological aspects of our work can be summarized as:

(iv) H-bond propensities tool can be applied for prediction of the most likely hydrogen bonds not only in crystals of small molecules, but also in ligand–receptor complexes.
(v) Molecular Voronoi polyhedra were for the first time applied for analysis of variance of contribution of intermolecular interactions to the total molecular area depending on some molecular conformation. Strength and shortcomings of this approach were discussed.

## Figures and Tables

**Table 1:** The most valuable torsion angles for Q1–Q8 rotation and their potentials.

Name of Dihedral Angle	Ima		HIma^+^	
	Angle (degree)	Rotation Potential (kcal/mol)	Angle (degree)	Rotation Potential (kcal/mol)
Q1	−17.99	4.84	−17.99	4.95
Q2	13.78	15.29	−2.22	16.61
Q3	−0.63	10.96	0.36	11.32
Q4	177.18	5.53	176.18	4.88
Q5	178.85	17.43	178.85	18.82
Q6	25.54	4.22	153.54	5.36
Q7	140.00	5.89	56.00	9.10
Q8	−165.11	7.00	−170.11	6.05

**Table 2:** Propensities of H-bonding in Ima and HIma^+^ molecules.

Donor	Acceptor	Ima	HIma^+^	Observed in CSD	Observed in PDB ^1^	Observed in PDB ^2^
Amine R_2_NH	N (pyridine)	0.5	0.39			
	O=C	0.41	0.34		Yes	
	N (pyrimidine)	0.21	0.15–0.22			
	N (piperidyl)	0.14–0.20	0.12			
	O (water)		0.32			Yes
Amide C(O)NH	N (pyridine)	0.44	0.34	Yes	Yes	
	O=C	0.35	0.28		Yes	Yes
	N (pyrimidine)	0.17	0.12–0.25	Yes		Yes
	N (piperidyl)	0.11–0.16	0.10			
	O (water)		0.27			Yes
Ammonium R_3_NH	N (pyridine)		0.43			
	O=C		0.37		Yes	
	N (pyrimidine)		0.17			
	N (piperidyl)		0.14			
	O (water)		0.36	Yes		Yes
Water H_2_O	N (pyridine)		0.7		Yes	Yes
	O=C		0.65		Yes	
	N (pyrimidine)		0.39–0.49		Yes	Yes
	N (piperidyl)		0.30		Yes	Yes
	O (water)		0.63		Yes	Yes

^1^ Extended conformation; ^2^ folded conformation.

**Table 3:** One-way analysis of variance (ANOVA) for π…π interactions.

	π…π (C…C only)			π…π (C…C and C…N)		
*p* value	<0.0001			<0.0001		
F_exp_	176.8			440.5		
F_crit_	4.2			4.2		
R^2^	0.95			0.98		
R_min_	0.94			0.97		
R_max_	1.00			0.99		
ANOVA	SS	DF	MS	SS	DF	MS
Treatment (between columns)	742.8	1	742.8	2552.5	1	2552.5
Residual (within columns)	105.0	25	4.2	144.9	25	5.8
Total	847.9	26		2697.3	26	

## Supplementary Materials

Supplementary materials can be found at https://www.mdpi.com/1422-0067/21/23/8970/s1.

## Abbreviations

API	Active pharmaceutical ingredient
CSD	Cambridge Structural Database
DFT	Density Functional Theory
Ima	Imatinib
MVP	Molecular Voronoi Polyhedron
PDB	Protein Data Bank

## Appendix A

CSD codes taken for analysis of imatinib conformations: DUNTIQ, RAYQEP, XAVTOF-XAVTOF03, XEJLUW, XEJMEH.PDB codes taken for analysis of imatinib conformations: 1IEP, 1OPJ, 1T46, 1XBB, 2HYY, 2OIQ, 3FW1, 3GVU, 3HEC, 3K5V, 3MS9, 3MSS, 3OEZ, 3PYY, 4BKJ, 4CSV, 6HD4, 6HD6, 6JOL, 6NPE, 6NPU, 6NPV.

CSD codes taken for analysis of molecular Voronoi polyhedra: DUNTIQ, RAYQEP, XAVTOF, XAVTOF02, XEJLUW, XEJMEH. PDB codes taken for analysis of molecular Voronoi polyhedra: 1IEP (two independent molecules), 1OPJ (two independent molecules), 1T46, 3FW1, 3GVU, 3K5V (two independent molecules), 3MS9 (two independent molecules), 3MSS (four independent molecules), 3PYY (two independent molecules), 4BKJ (two independent molecules), 6NPV (two independent molecules).

## Appendix B

All statistical calculations were carried out using MS Excel for Office 365. The number of measured MVP volumes and areas is relatively low (*n* = 27), thus, the assumption about their normal distribution was verified by comparison of the skewness (*α*) and kurtosis (*∊*) with their standard errors (*S_α_* and *S_ε_*) calculated as the following:

(A1) $$\alpha =\frac{\sqrt{n\left(n-1\right)}}{n-2}\cdot \frac{m_{3}}{m_{2}^{3/2}}$$

(A2) $$\epsilon =\frac{\left(n-1\right)\left(\left(n+1\right)\left(\frac{m_{4}}{m_{2}^{2}}-3\right)+6\right)}{\left(n-2\right)\left(n-3\right)}$$

(A3) $$\;S_{\alpha }=\sqrt{\frac{6n\left(n-1\right)}{\left(n-2\right)\left(n+1\right)\left(n+3\right)}}$$

(A4) $$\;S_{\varepsilon }=\sqrt{\frac{24n{\left(n-1\right)}^{2}}{\left(n-3\right)\left(n-2\right)\left(n+3\right)\left(n+5\right)}}$$

where *m*_2_, *m*_3_, and *m*_4_ are moments of inertia calculated using formula:

(A5) $$\;m_{k}=\frac{1}{n}\overset{n}{\underset{i=1}{\sum }}{\left(x_{i}-\bar{x}\right)}^{k}$$

For MVP volumes, *α* = −0.11 and *∊* = −1.34, and for MVP areas *α* = 0.46 and *∊* = −0.72. For both datasets, *S_α_* = 0.45 and *S_ε_* = 0.87. Although both datasets demonstrate nonzero skewness and kurtosis, we cannot reject the normality hypothesis as |*α*| < 3*S_α_* and |*∊*| < 5*S_ε_*. For G_3_ values *α* = 1.72 and *∊* = 1.74; and *α* = 1.72 > 3*S_α_* =1.34 so the second normalized moment for extended and folded conformations deviate from normal distribution.

A confidence interval [*x_min_*; *x_max_*] was calculated at the confidence level *p* = 0.95 using a random Student’s variable η as

(A6) $$\eta =\left|\tau \right|\frac{\sqrt{n-2}}{\sqrt{n-1-\tau^{2}}}$$

where $\left|\tau \right|=\left(\bar{x}-x_{min}\right)/\sigma =\left(x_{max}-\bar{x}\right)/\sigma$; $\bar{x}$ and *σ* are the mean and standard deviation, respectively.
