# Supplementary material for: Intermolecular Interactions in Crystal Structures of Imatinib-Containing Compounds
Source: Int J Mol Sci. 2020 Nov 26;21(23):8970. doi: 10.3390/ijms21238970 (PMC7731260; doi:10.3390/ijms21238970)
Supplement: Supplementary file 1 [file ijms-21-08970-s001.pdf]

Optimized structure of folded conformer of Ima.

C	-7.009330000	0.228962000	1.966269000
H	-6.783816000	0.596963000	2.974511000
H	-6.995475000	-0.878973000	2.003747000
C	-5.927850000	0.707097000	1.000458000
H	-4.963156000	0.278485000	1.290585000
H	-5.843133000	1.809541000	1.068464000
C	-7.549120000	0.801521000	-0.766860000
H	-7.560805000	1.908668000	-0.795408000
H	-7.776362000	0.438760000	-1.776531000
C	-8.628134000	0.319148000	0.200719000
H	-8.715301000	-0.783314000	0.120939000
H	-9.594413000	0.750050000	-0.088474000
C	-9.362345000	0.361362000	2.502173000
H	-9.116485000	0.740227000	3.500517000
H	-10.314427000	0.807749000	2.193830000
H	-9.506958000	-0.734694000	2.578270000
C	-5.190488000	0.642457000	-1.315260000
H	-5.585652000	0.451585000	-2.322891000
H	-4.947149000	1.723136000	-1.279258000
C	-3.918697000	-0.165892000	-1.133078000
C	-2.662979000	0.438642000	-1.247603000
H	-2.597498000	1.507742000	-1.436914000
C	-1.492505000	-0.311154000	-1.130965000
H	-0.537411000	0.191894000	-1.262628000
C	-1.553147000	-1.689850000	-0.887050000
C	-2.812806000	-2.298852000	-0.785328000
H	-2.852747000	-3.370036000	-0.617344000
C	-3.976828000	-1.547735000	-0.900235000
H	-4.949510000	-2.019675000	-0.798865000
C	-0.348244000	-2.580680000	-0.751354000
C	2.103032000	-2.476354000	-0.185326000
C	2.412932000	-3.831548000	-0.347408000
H	1.646458000	-4.531839000	-0.644473000
C	3.723611000	-4.243560000	-0.110248000
H	3.965913000	-5.296554000	-0.233504000
C	4.741163000	-3.370527000	0.275056000
C	4.409551000	-2.002529000	0.427086000
C	3.100485000	-1.569619000	0.200382000
H	2.869486000	-0.518917000	0.317823000
C	6.144033000	-3.875938000	0.515777000
H	6.464893000	-3.747681000	1.560544000
H	6.208693000	-4.945037000	0.294915000
H	6.883093000	-3.365111000	-0.116244000
C	5.538892000	0.234939000	0.855913000
C	6.842725000	2.001046000	1.434635000
H	7.769390000	2.368337000	1.873732000
C	5.897060000	2.892908000	0.925366000
H	6.073885000	3.961224000	0.932362000
C	4.742086000	2.328239000	0.374317000
C	3.663260000	3.149457000	-0.226596000
C	2.830263000	2.619162000	-1.222245000
H	2.984033000	1.596991000	-1.552884000
C	1.838184000	3.422947000	-1.773348000
H	1.185669000	3.049930000	-2.557525000
C	1.702741000	4.732267000	-1.306087000
H	0.941059000	5.391057000	-1.719885000

C	3.425745000	4.474310000	0.171753000
H	4.019870000	4.921045000	0.966955000
N	-8.317626000	0.739542000	1.564268000
N	-6.240505000	0.284329000	-0.366566000
N	0.814416000	-1.937210000	-0.386850000
N	5.435583000	-1.133213000	0.841920000
N	4.563531000	0.995513000	0.340614000
N	6.690654000	0.681519000	1.413439000
N	2.473736000	5.256323000	-0.345400000
O	-0.425765000	-3.790160000	-0.942359000
H	6.269293000	-1.568317000	1.212041000
H	0.726425000	-0.960929000	-0.142986000

Total energy = -1582.2146072 a.u.

Optimized structure of extended conformer of Ima.

C	7.721634000	-1.954921000	1.659710000
H	7.330897000	-2.667497000	2.396238000
H	7.792727000	-0.966138000	2.156026000
C	6.747673000	-1.853880000	0.488234000
H	5.790268000	-1.457777000	0.841789000
H	6.565186000	-2.867733000	0.080489000
C	8.582641000	-1.434255000	-1.000330000
H	8.508029000	-2.425744000	-1.488439000
H	8.974904000	-0.726282000	-1.740281000
C	9.554602000	-1.531773000	0.173962000
H	9.741345000	-0.513584000	0.571125000
H	10.513093000	-1.930778000	-0.179774000
C	9.960347000	-2.598774000	2.303351000
H	9.550258000	-3.314444000	3.024719000
H	10.906684000	-3.001424000	1.925020000
H	10.180922000	-1.656799000	2.843803000
C	6.342590000	-0.757348000	-1.643713000
H	6.886890000	-0.227088000	-2.437722000
H	6.009379000	-1.717060000	-2.086805000
C	5.127872000	0.066459000	-1.256307000
C	3.856154000	-0.264676000	-1.734306000
H	3.731543000	-1.141703000	-2.365564000
C	2.746065000	0.520654000	-1.422653000
H	1.780810000	0.253376000	-1.845964000
C	2.884626000	1.656975000	-0.614574000
C	4.162279000	1.997971000	-0.146768000
H	4.266132000	2.892003000	0.459328000
C	5.265351000	1.210910000	-0.457060000
H	6.249321000	1.465837000	-0.075030000
C	1.747278000	2.566595000	-0.237885000
C	-0.756535000	2.550601000	0.024141000
C	-0.943422000	3.909164000	0.301911000
H	-0.097637000	4.580029000	0.328288000
C	-2.239156000	4.360815000	0.548400000
H	-2.387693000	5.416127000	0.765123000
C	-3.355437000	3.525216000	0.532558000
C	-3.145069000	2.152615000	0.249980000
C	-1.854694000	1.678774000	-0.001631000
H	-1.719186000	0.626936000	-0.217491000
C	-4.734286000	4.077283000	0.809473000
H	-5.202908000	3.614182000	1.689868000
H	-4.682943000	5.152177000	1.003817000

H	-5.420723000	3.936944000	-0.038034000
C	-4.459111000	-0.017803000	0.012400000
C	-3.708547000	-2.101080000	-0.463026000
H	-2.864387000	-2.753122000	-0.683282000
C	-4.995090000	-2.615932000	-0.387920000
H	-5.179775000	-3.674341000	-0.522322000
C	-6.022911000	-1.699291000	-0.099212000
C	-7.445529000	-2.107342000	0.009106000
C	-7.933968000	-3.297174000	-0.546559000
H	-7.278702000	-3.958226000	-1.106201000
C	-9.281162000	-3.613944000	-0.399667000
H	-9.691160000	-4.524764000	-0.825538000
C	-10.101295000	-2.728689000	0.300560000
H	-11.160404000	-2.943962000	0.432356000
C	-8.367209000	-1.291703000	0.689216000
H	-8.022455000	-0.361152000	1.130712000
N	9.027049000	-2.424622000	1.202129000
N	7.278150000	-0.957601000	-0.541172000
N	0.502397000	1.976067000	-0.252251000
N	-4.281506000	1.323533000	0.238195000
N	-5.749528000	-0.407143000	0.103744000
N	-3.411755000	-0.811777000	-0.272825000
N	-9.661215000	-1.584374000	0.840515000
O	1.943518000	3.738193000	0.069050000
H	-5.162361000	1.778654000	0.432363000
H	0.484501000	0.976536000	-0.397711000

Total energy = -1582.2227591 a.u.

Optimized structure of protonated folded conformer of Ima.

C	7.479103000	-0.717880000	-1.303870000
H	7.543927000	-1.139146000	-2.310239000
H	7.456391000	-1.523955000	-0.566912000
C	6.279954000	0.207025000	-1.142862000
H	5.374307000	-0.391063000	-1.276361000
H	6.286191000	0.971240000	-1.948394000
C	7.460857000	1.614354000	0.387121000
H	7.532141000	2.456954000	-0.332403000
H	7.439360000	2.055224000	1.389073000
C	8.716242000	0.752498000	0.290418000
H	8.709369000	-0.036789000	1.045676000
H	9.630846000	1.343787000	0.382490000
C	9.988605000	-0.806115000	-1.211380000
H	9.989950000	-1.242632000	-2.211509000
H	10.876795000	-0.188490000	-1.068087000
H	9.954955000	-1.595050000	-0.458686000
C	5.020215000	1.501507000	0.513729000
H	5.165271000	1.948913000	1.504810000
H	4.834285000	2.332559000	-0.189214000
C	3.830120000	0.566662000	0.534706000
C	3.833768000	-0.567356000	1.362370000
H	4.699231000	-0.770635000	1.987782000
C	2.747883000	-1.433634000	1.379761000
H	2.750187000	-2.324589000	1.998771000
C	1.617492000	-1.178576000	0.588596000
C	1.611944000	-0.049384000	-0.238346000
H	0.773231000	0.149962000	-0.899829000
C	2.711445000	0.811507000	-0.267117000

H	2.695181000	1.677742000	-0.924624000
C	0.503552000	-2.197437000	0.641403000
C	-1.969521000	-2.412441000	0.238477000
C	-2.087517000	-3.795074000	0.405959000
H	-1.221584000	-4.395308000	0.642856000
C	-3.351737000	-4.367809000	0.266552000
H	-3.450930000	-5.442557000	0.396845000
C	-4.496615000	-3.628180000	-0.027753000
C	-4.354841000	-2.227179000	-0.189496000
C	-3.095891000	-1.633746000	-0.056195000
H	-3.009661000	-0.561967000	-0.180798000
C	-5.839015000	-4.306262000	-0.165124000
H	-6.256017000	-4.208983000	-1.178285000
H	-5.751127000	-5.376557000	0.039784000
H	-6.581543000	-3.899072000	0.534237000
C	-5.762156000	-0.148437000	-0.595992000
C	-7.301992000	1.424527000	-1.151758000
H	-8.300549000	1.659283000	-1.516952000
C	-6.415376000	2.444389000	-0.799032000
H	-6.706963000	3.485814000	-0.855939000
C	-5.161550000	2.044639000	-0.328488000
C	-4.133055000	3.017647000	0.111963000
C	-3.201945000	2.676909000	1.103068000
H	-3.241592000	1.689232000	1.551509000
C	-2.263825000	3.622317000	1.503771000
H	-1.541091000	3.399462000	2.283446000
C	-2.277807000	4.877833000	0.891986000
H	-1.563742000	5.645056000	1.187090000
C	-4.043991000	4.305452000	-0.439765000
H	-4.720865000	4.605863000	-1.237599000
N	8.766768000	0.052052000	-1.056304000
N	6.277034000	0.795708000	0.187936000
N	-0.739659000	-1.714644000	0.330080000
N	-5.508768000	-1.497154000	-0.514175000
N	-4.833215000	0.742915000	-0.226424000
N	-7.001783000	0.133683000	-1.060810000
N	-3.142552000	5.220035000	-0.070632000
O	0.746630000	-3.359573000	0.950744000
H	8.810579000	0.788992000	-1.771210000
H	-6.321073000	-2.037333000	-0.779021000
H	-0.819374000	-0.714076000	0.211828000

Total energy = -1582.6557166 a.u.

Optimized structure of protonated extended conformer of Ima.

C	8.571354000	-0.242218000	1.368012000
H	8.629447000	-0.030727000	2.438694000
H	8.601404000	0.693268000	0.804676000
C	7.338050000	-1.060096000	1.008306000
H	6.455780000	-0.457987000	1.242408000
H	7.291368000	-1.967186000	1.647067000
C	8.494984000	-2.193581000	-0.750334000
H	8.512034000	-3.163546000	-0.210189000
H	8.479325000	-2.427486000	-1.819805000
C	9.783554000	-1.427598000	-0.465580000
H	9.829171000	-0.506124000	-1.050564000
H	10.674023000	-2.031580000	-0.657654000
C	11.083776000	-0.255071000	1.333879000

H	11.079176000	-0.023900000	2.400346000
H	11.948052000	-0.873309000	1.085513000
H	11.102341000	0.666982000	0.751156000
C	6.065189000	-1.937778000	-0.893890000
H	6.217124000	-2.188929000	-1.950974000
H	5.820962000	-2.880134000	-0.372602000
C	4.921028000	-0.957185000	-0.752001000
C	5.010104000	0.323013000	-1.321300000
H	5.908906000	0.606342000	-1.862878000
C	3.966373000	1.229996000	-1.188572000
H	4.034912000	2.229653000	-1.604452000
C	2.793266000	0.874449000	-0.505970000
C	2.701944000	-0.401007000	0.062751000
H	1.826729000	-0.688816000	0.638928000
C	3.760148000	-1.305073000	-0.055177000
H	3.678384000	-2.287461000	0.404454000
C	1.729292000	1.938879000	-0.378365000
C	-0.753058000	2.197704000	-0.077463000
C	-0.799207000	3.587969000	0.057000000
H	0.107670000	4.173427000	0.027356000
C	-2.046736000	4.190143000	0.219958000
H	-2.090830000	5.271429000	0.323949000
C	-3.241513000	3.472840000	0.253715000
C	-3.171203000	2.063144000	0.114662000
C	-1.930460000	1.439224000	-0.047872000
H	-1.901323000	0.361778000	-0.150570000
C	-4.561489000	4.185356000	0.431107000
H	-5.093132000	3.860015000	1.336524000
H	-4.405832000	5.263710000	0.521226000
H	-5.238191000	4.028289000	-0.420774000
C	-4.691294000	0.021068000	0.041370000
C	-4.151314000	-2.160852000	-0.221082000
H	-3.376311000	-2.912963000	-0.362074000
C	-5.486544000	-2.529987000	-0.164204000
H	-5.782783000	-3.564675000	-0.282177000
C	-6.420897000	-1.493050000	0.021192000
C	-7.880447000	-1.739744000	0.103082000
C	-8.418323000	-3.000426000	0.395678000
H	-7.770400000	-3.847641000	0.600743000
C	-9.800105000	-3.153801000	0.450420000
H	-10.247432000	-4.115512000	0.682207000
C	-10.604484000	-2.038920000	0.210090000
H	-11.689183000	-2.122302000	0.247500000
C	-8.789440000	-0.689543000	-0.117407000
H	-8.407303000	0.300894000	-0.346889000
N	9.830593000	-1.008011000	0.993447000
N	7.343594000	-1.374751000	-0.412281000
N	0.451454000	1.467967000	-0.236748000
N	-4.382734000	1.356799000	0.149171000
N	-6.015481000	-0.222771000	0.118602000
N	-3.722879000	-0.897087000	-0.117051000
N	-10.116628000	-0.823315000	-0.070872000
O	2.040877000	3.125290000	-0.410715000
H	9.825154000	-1.872135000	1.549615000
H	-5.218137000	1.912713000	0.268247000
H	0.321158000	0.472648000	-0.355190000

Total energy = -1582.6559500 a.u.

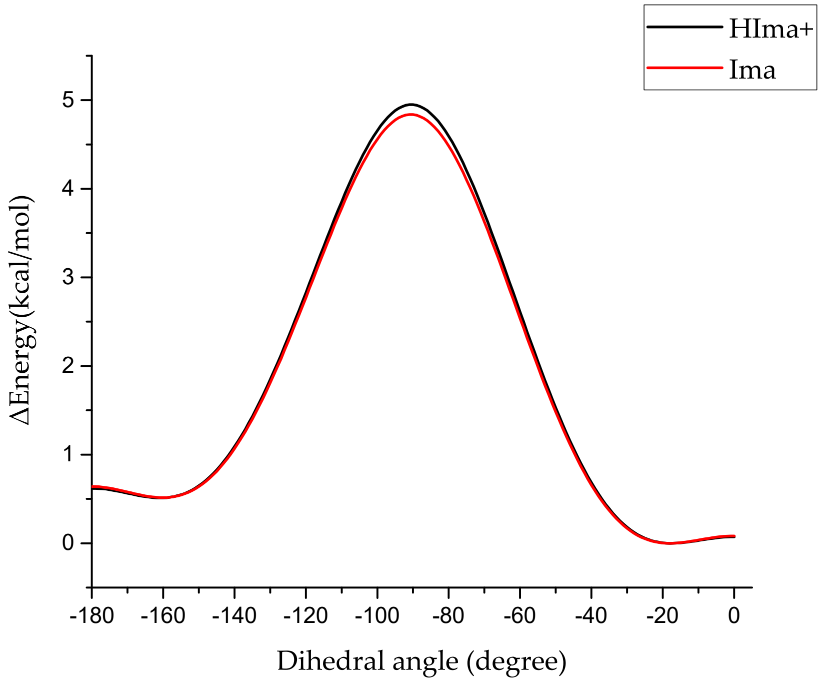

Figure S1. The dependence of the energy by the dihedral angle Q1 in the Ima molecule (red line) and the HIma+ molecule (black line).

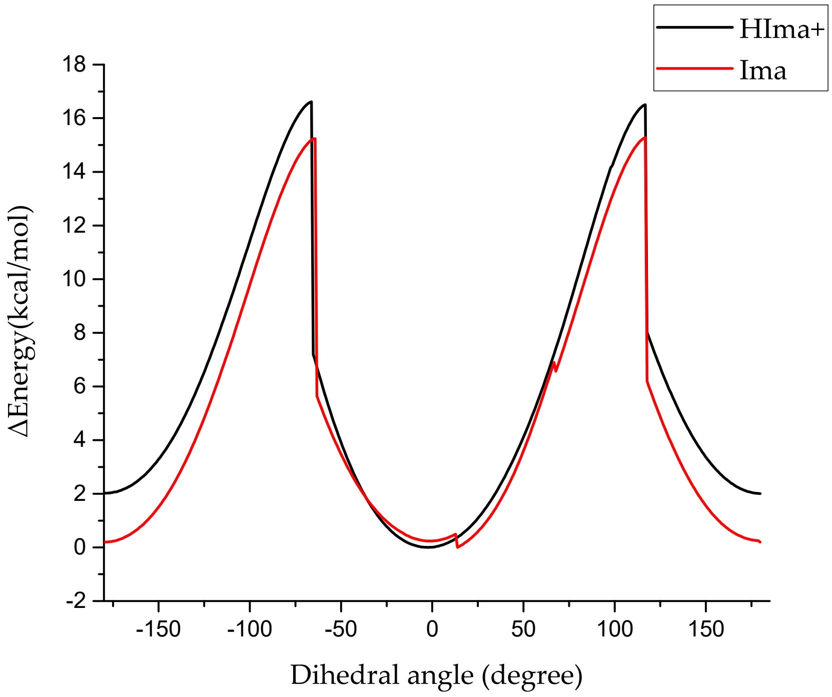

Figure S2. The dependence of the energy by the dihedral angle Q2 in the Ima molecule (red line) and the HIma+ molecule (black line).

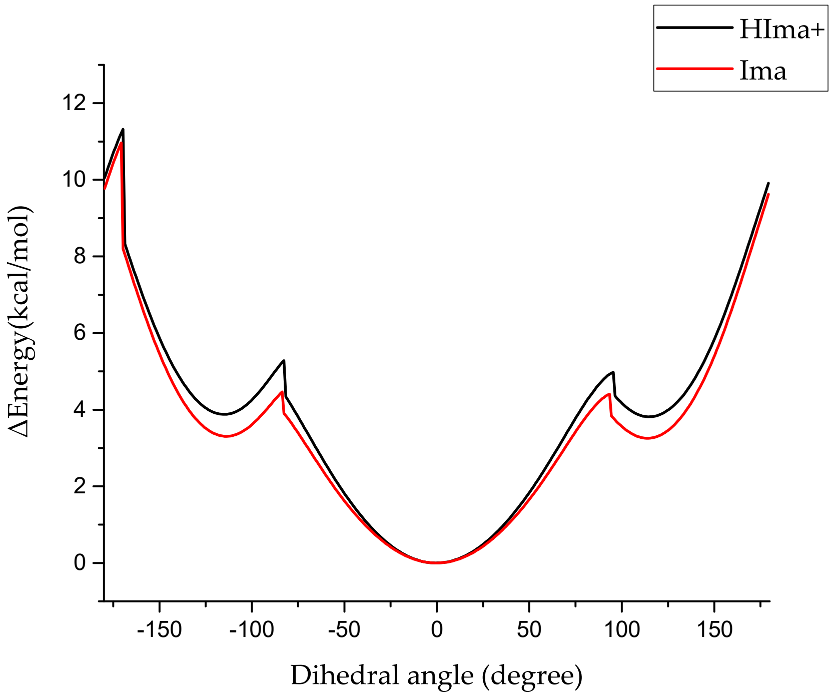

Figure S3. The dependence of the energy by the dihedral angle Q3 in the Ima molecule (red line) and the HIma+ molecule (black line).

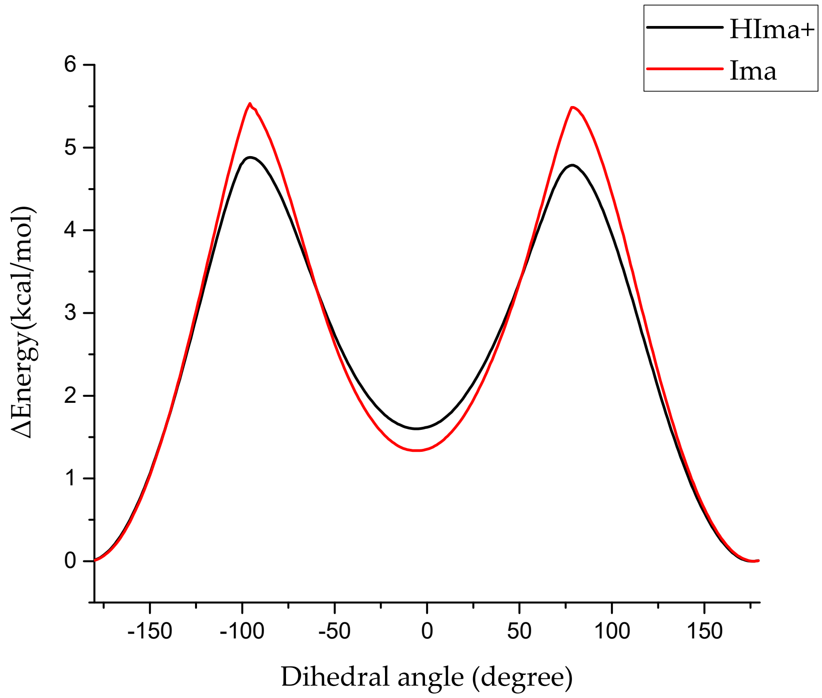

Figure S4. The dependence of the energy by the dihedral angle Q4 in the Ima molecule (red line) and the HIma+ molecule (black line).

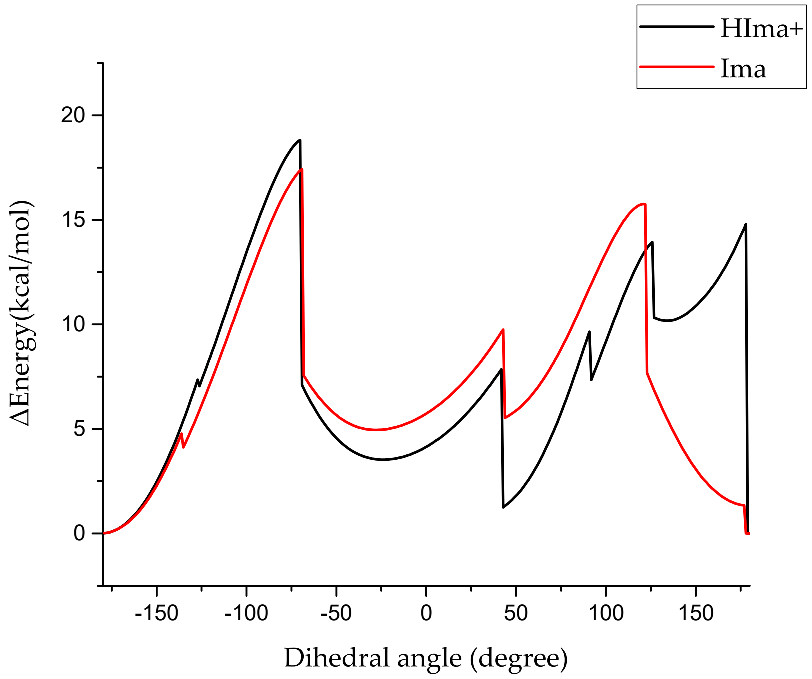

Figure S5. The dependence of the energy by the dihedral angle Q5 in the Ima molecule (red line) and the HIma+ molecule (black line).

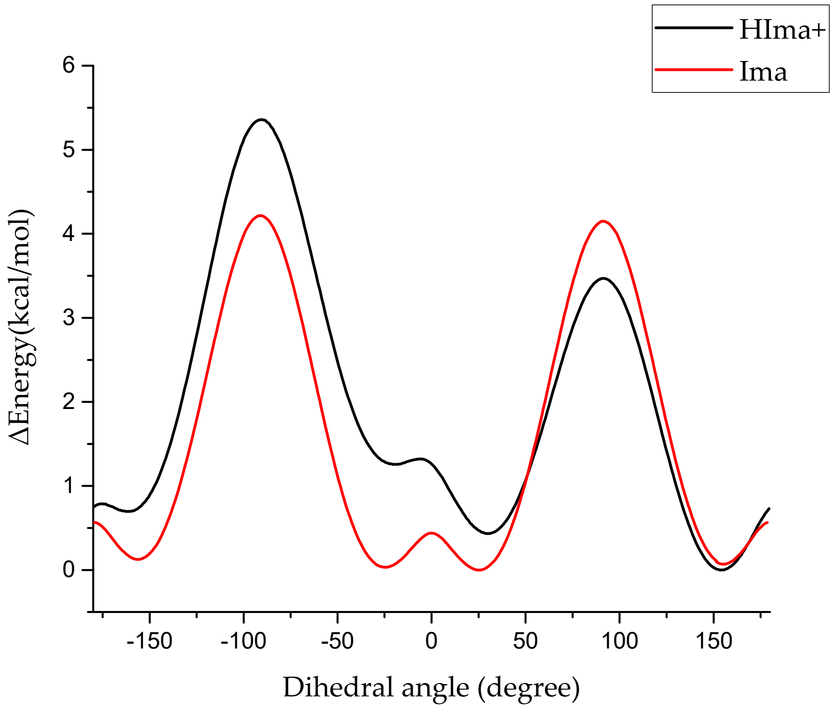

Figure S6. The dependence of the energy by the dihedral angle Q6 in the Ima molecule (red line) and the HIma+ molecule (black line).

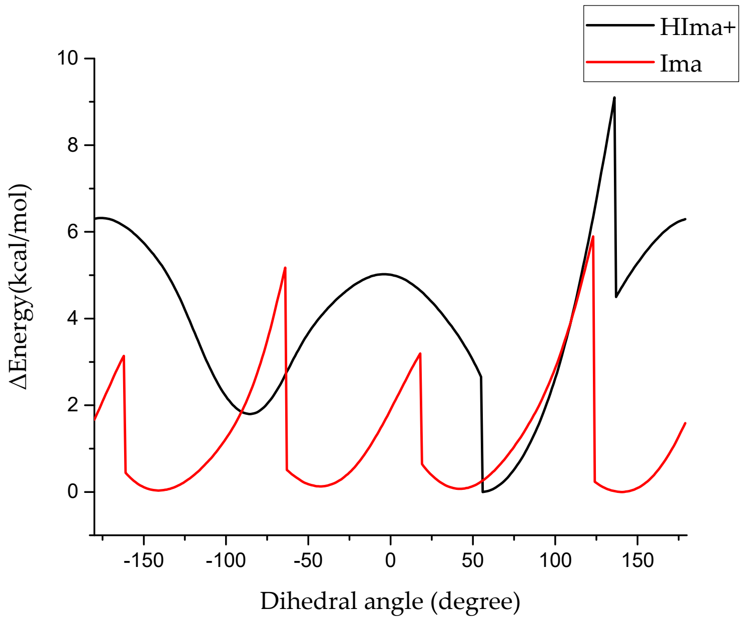

Figure S7. The dependence of the energy by the dihedral angle Q7 in the Ima molecule (red line) and the HIma+ molecule (black line). Such a difference between the black line (HIMA+) and the red line (IMA) is a result of the lack of opportunity to change the conformation of the methyl-pipyridine fragment in the protonated IMA (HIMA +) molecule.

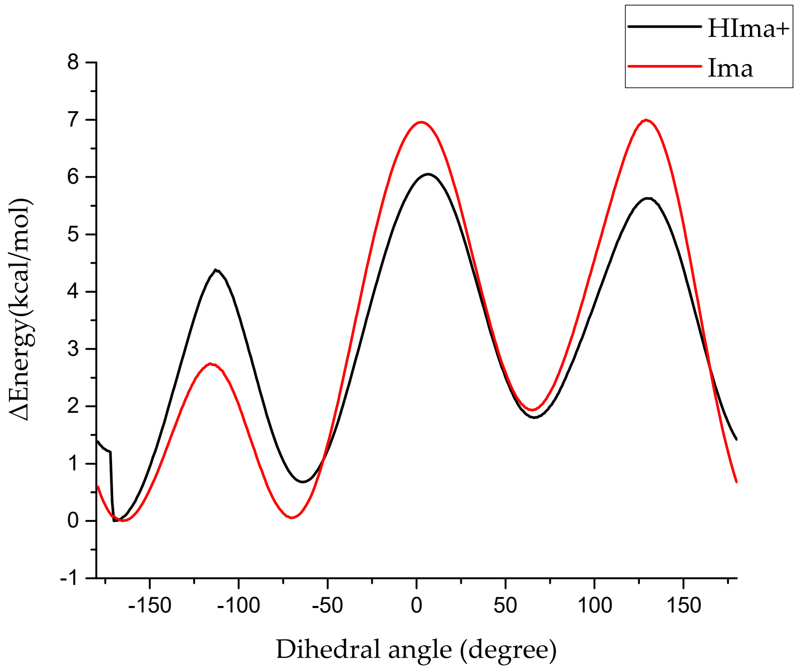

Figure S8. The dependence of the energy by the dihedral angle Q8 in the Ima molecule (red line) and the HIma+ molecule (black line).

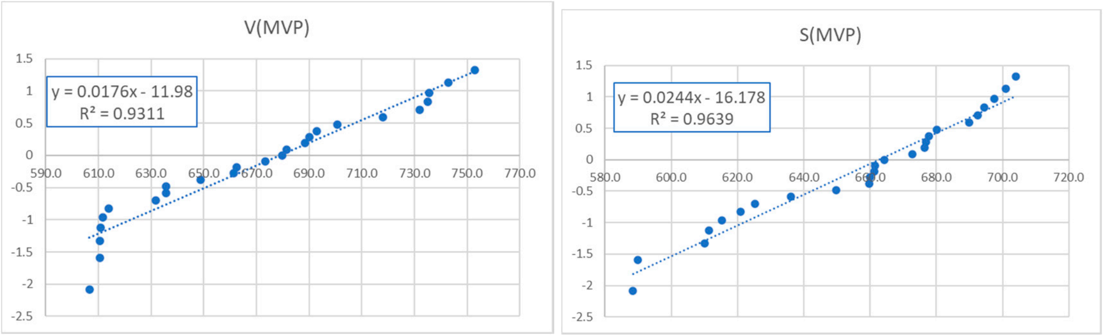

Figure S9. Normal probability plots for molecular volume (V_{MVP}) and area (S_{MVP}) of Imatinib in ligand – receptor complexes and salts.

Table S1. Molecular conformations of Imatinib in crystal structures taken from CSD and PDB as observed from torsion angles Q1-Q8 [°][1].

Refcode	Q1	Q2	Q3	Q4	Q5	Q6	Q7	Q8
Extended								
1IEP	168 – 170	179	97 – 98	180	168	-156 – -165	-130	179 – 180
1OPJ	172 – 176	178 – 179	95 – 97	179 – 180	167	-160 – -167	-127 – -131	175 – 177
1T46	176	172	85	177	177	-164	-126	165
2HYY	-175 – -179	172 – 177	96 – 102	-171	166	-161 – -165	-135 – -137	-173 – -177
2OIQ	-175	179	95	179	168	-163	-129	179
3GVU	153 – 175	151 – 171	102 – 103	177 – 179	173 – 178	-139 – -157	45 – 89	-158 – -178
3HEC	163	169	81	-162	174	-149	-175	-155
3K5V	173 – 176	174 – 175	98 – 103	177 – 179	173 – 175	-151 – -159	-128 – -135	-173
3MS9	177 – 178	177 – 179	97 – 104	-169 – -174	172	-159 – -161	-134 – -138	174 – 176
3MSS	174 – 176	174 – 179	93 – 102	-172 – -179	172 – 174	-154 – -157	128 – 134	-168 – -171
3OEZ	177 – 178	173 – 178	102 – 104	-173 – -179	172 – 174	-154 – -169	-117 – -126	-174 – -179
4BKJ	167 – 168	175 – 179	91 – 98	-173 – -176	175 – 176	-152 – -156	-137	-175 – -178
4CSV	-179	-177	89	178	169	-164	85	-159
6HD4	171 – 175	172 – 173	98 – 104	-176 – -177	174 – 175	-154 – -155	-135	-176 – -177
6HD6	173 – 176	171 – 176	94 – 108	-176 – -178	174 – 175	-159	-135 – -137	-166 – -175
6JOL	-171	173	79	174	178	-155	-128	-175
6NPE	170 – 174	175 – 179	93 – 97	-173	164 – 165	-163 – -168	-122 – -129	-160 – -173
6NPU	172 – 174	-179	96 – 99	166 – 179	176 – 177	-156 – -159	-128 – -132	-162 – -169
6NPV	172 – 178	177 – 179	95 – 97	175 – 178	156 – 174	-155 – -156	-132 – -134	-177 – -178
XAVTOF	141	153	78	-165	176	-158	-137	-167
XAVTOF01	139	-154	-77	-163	176	-156	137	-165
Folded								
1XBB	178	1	-2	-176	-167	-143	151	95
3FW1	-179	4	-33	-174	-169	139	-127	174
DUNTIQ	-166	18	17	23	-171	-156	107	-179
RAYQEP	145	1	-9	1	-175	-149	159	165
XAVTOF02	-165	4	113	129	-179	144	-160	168
XAVTOF03	-166	3	114	129	-179	145	157	146
XEJLUW	-161	0	29	-133	-161	-150	104	176
XEJMEH	-160	-1	30	-133	164	-149	103	-177

[1] Notation of angles Q1-Q8 is visualized on Scheme 1.

Table S2. Descriptors of molecular Voronoi polyhedra of Imatinib in ligand - receptor complexes and salts.[a.]

	V_{MVP}, Å^3	R_{sd}, Å	G_3	S_{MVP}, Å^2	Contribution of various intermolecular interactions to the total molecular area, Å^2													
					H...H	C...H	O...H	N...H	Cl…H	S…H	C…C	C…O	C…S	C…N	N…O	N…S	O…O	N…N
PDB entries																		
1IEP	735,1	5,6	0,3	707,1	401,8	118,4	103,2	37,0	23,6	5,7	4,8	6,1	1,7	2,1	0,2	2,0	0,7	
	735,4	5,6	0,3	676,4	381,7	121,9	98,9	39,1	14,7	6,4	5,9	3,8		3,1	0,0		0,8	0,0
1OPJ	718,1	5,6	0,3	694,4	388,4	127,5	94,7	44,1	22,1	5,9	4,9	3,1	0,0	0,1	3,5		0,2	
	752,9	5,6	0,3	724,4	421,7	120,3	96,0	43,8	17,5	6,3	4,9	7,7		0,3	5,5		0,6	
1T46	610,7	5,3	0,2	636,0	357,8	118,1	104,9	39,0		6,1	0,1	4,0		0,7	4,5		0,8	
3GVU	662,4	5,4	0,3	661,2	374,8	119,8	112,5	35,9		6,4	2,0	4,4		0,4	4,4		0,7	
3K5V	661,4	5,4	0,3	676,9	361,5	124,1	133,3	38,7	0,1	6,5	3,6	2,4		2,5	3,6		0,6	
	692,8	5,5	0,3	701,0	399,7	115,7	120,1	34,8		6,5	4,1	9,1		2,4	7,7		1,0	
3MS9	688,5	5,5	0,3	661,5	370,7	120,6	107,4	36,2	10,4	6,3	4,3	3,9		1,4	0,1		0,3	
	673,2	5,4	0,3	664,4	362,0	116,1	116,1	36,4	9,5	6,0	4,2	9,0		1,8	2,4		0,8	
3MSS	700,7	5,5	0,3	677,8	387,7	120,4	114,3	34,1		6,4	4,2	4,8		1,6	3,6		0,7	
	681,4	5,5	0,3	672,8	369,3	110,9	131,5	31,2		6,7	4,4	11,1		1,7	5,2		0,9	
	679,8	5,5	0,3	659,9	360,4	121,8	124,8	36,6		6,3	4,5	3,2		1,8	0,0		0,3	
	690,1	5,5	0,3	692,5	410,5	120,6	107,6	37,1		6,1	4,9	3,7		1,8	0,0		0,3	
3PYY	731,8	5,6	0,3	680,1	407,5	118,6	103,5	35,7		6,3	3,9	1,8	0,0	1,8	0,3	0,1	0,6	
	743,0	5,6	0,3	704,0	421,7	121,0	111,4	37,1		6,0	1,5	1,6	0,2	1,8	0,9	0,5	0,5	
4BKJ	648,7	5,4	0,2	649,8	354,6	96,2	124,6	37,3		11,7	5,9	8,3	4,1	1,0	2,6	0,2	0,8	2,5
	635,8	5,3	0,2	660,1	368,3	104,6	118,5	42,0		10,9	6,0	4,7	2,7	0,7	1,0	0,2	0,6	
6NPV	755,8	5,7	0,3	690,0	398,9	120,5	117,2	38,4		6,3	3,7	2,1		1,2	0,7		0,9	
	753,3	5,6	0,3	697,4	392,0	122,6	129,2	38,3		6,4	4,1	2,0		1,8			0,9	
3FW1	631,8	5,3	0,2	625,2	314,9	101,0	125,5	26,2		6,6	21,4	2,9	1,61	12,7	8,9		0,6	2,9
CSD entries																		
XAVTOF	611,6	5,3	0,2	615,2	275,6	169,2	86,4	71,1		0,5	8,0	0,3		3,8			0,4	

XAVTOF02	613,9	5,3	0,2	611,3	295,4	128,4	93,8	60,8		0,4	14,9	1,8		15,0	0,1		0,7	0,1
RAYQEP	635,6	5,3	0,2	610,1	233,6	123,2	151,3	52,0			19,3	9,2		11,5	9,2		0,0	2,4
XEJLUW	610,9	5,3	0,1	590,0	281,6	131,7	92,3	57,8			13,7			12,6				
XEJMEH	606,6	5,3	0,1	588,4	284,6	134,4	82,4	60,8		0,7	14,0			11,7				
DUNTIQ	610,5	5,3	0,2	620,9	174,3	135,0	205,6	58,6			18,0	15,0		10,9	1,1			2,32

[a] V_{MVP} - the volume of Imatinib in a crystals; R_{SD} - the radius of the sphere of a V_{MVP} volume; G_3 - the second normalized moment that estimates the molecular shape; S_{MVP} - the area of the molecular Voronoi polyhedron.

Table S3. One-way analysis of variance for hydrophobic and hydrophilic interactions.

	Hydrophobic			Hydrophilic		
P value	< 0.0001			0.125		
F_{exp}	38.2			2.5		
F_{crit}	4.2			4.2		
R^2	0.80			0.14		
R_{min}	0.74			0.00		
R_{max}	0.96			0.28		
ANOVA	SS	df	MS	SS	df	MS
Treatment (between columns)	33740.7	1	33740.7	1250.8	1	1250.8
Residual (within columns)	22077.6	25	883.1	12433.1	25	497.3
Total	55818.3	26		13683.9	26	

Table S4. One-way analysis of variance for interactions sensitive to a molecular conformation.

	H…H			N…H			C…N		
P value	< 0.0001			0.004			< 0.0001		
F_{exp}	46.5			9.8			499.4		
F_{crit}	4.2			4.2			4.2		
R^2	0.83			0.49			0.98		
R_{min}	0.78			0.22			0.96		
R_{max}	0.88			0.71			1.00		
ANOVA	SS	df	MS	SS	df	MS	SS	df	MS
Treatment (between columns)	62056.5	1	62056.5	843.3	1	843.3	541.9	1	541.9
Residual (within columns)	33379.3	25	1335.2	2143.8	25	85.8	27.1	25	1.1
Total	95435.8	26		2987.2	26		569.1	26	

Table S5. One-way analysis of variance for interactions insensitive to a molecular conformation.

	C…H			O…H			O…H[1]		
P value	0.396			0.252			0.079		
F_{exp}	0.7			1.4			3.4		
F_{crit}	4.2			4.2			4.2		
R^2	0.03			0.04			0.13		
R_{min}	0.00			0.00			0.00		
R_{max}	0.09			0.11			0.26		
ANOVA	SS	df	MS	SS	df	MS	SS	df	MS
Treatment (between columns)	126.8	1	126.8	785.8	1	785.8	629.2	1	629.2
Residual (within columns)	4251.1	25	170.0	14284.8	25	571.4	4280.4	23	186.1
Total	4377.9	26		15070.6	26		4909.5	24	

[1] Data analyzed without 2,4,6-trinitrophenolate-containing salts {RAYQEP} and {DUNTIQ}.
